# Supplementary material for: New inhibitors of the Pseudomonas aeruginosa enzyme, PqsE, and methods assessing their potential to induce a conformational change via active site binding
Source: mSphere. 2026 Apr 20;11(5):e00826-25. doi: 10.1128/msphere.00826-25 (PMC13203959; doi:10.1128/msphere.00826-25)
Supplement: Supplemental information — Supplemental figures and table. [file msphere.00826-25-s0001.pdf]

Supporting Information for

New inhibitors of the *Pseudomonas aeruginosa* enzyme, PqsE, and methods assessing their potential to induce a conformational change via active site binding

Samantha B. Orr[1*], Hannah A. Jones[1*], Margaret G. O'Hara[1], Kaitlyn R. Smith[1], Isabelle R. Taylor[1#]

[1]Department of Chemistry, William & Mary, Williamsburg, VA 23185, USA

*Authors contributed equally

[#]To whom correspondence should be addressed. Email: irtaylor@wm.edu

This PDF file includes:

Figure S1
Figure S2
Figure S3
Figure S4
Table S1

Supplementary Figures

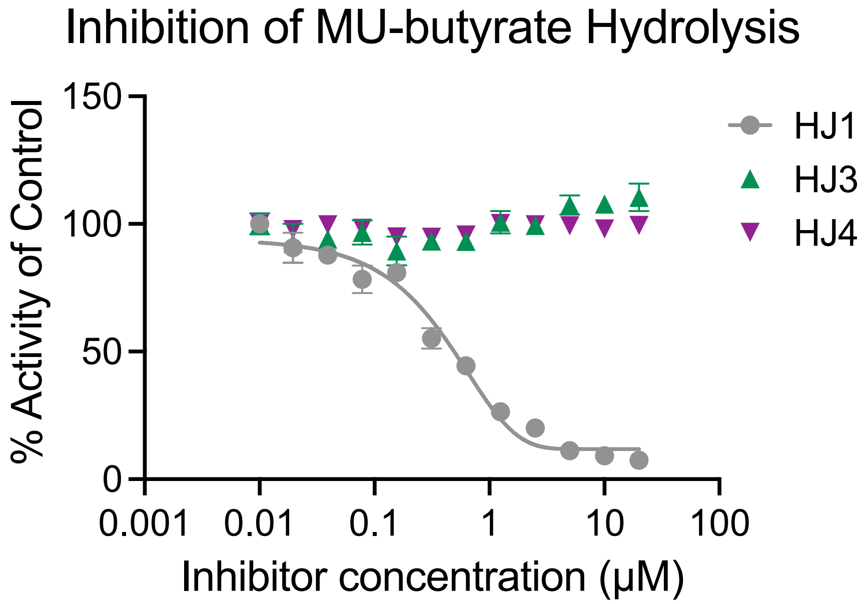

Figure S1: HJ2 and HJ3 do not inhibit PqsE enzymatic activity. Inhibition of PqsE-catalyzed MU-butyrate hydrolysis is shown. For comparison, HJ1 inhibits PqsE esterase activity with an IC_{50} of ~400 nM, whereas the chlorinated and brominated derivatives, HJ3 (green triangles) and HJ4 (purple upside-down triangles) show no inhibitory activity. Background fluorescence was measured for the compound dilution series plus MU-butyrate in the absence of PqsE and was subtracted from the values plotted prior to normalization. The data were normalized so that DMSO-treated PqsE is 100 % activity. Data shown are the average of technical triplicates and error bars represent standard deviation.

A T_m of PqsE-HJ complexes

	DMSO	HJ1	HJ2	HJ5
WT	66.9	70.5	67.7	68.8
E280A	66.5	68.8	67.1	67.7
E182W	73.4	74.4	73.9	75.5
E182W/E280A	69.8	72.4	70.0	71.9
S285W	66.7	66.8	66.8	67.5
S285A	67.3	68.1	67.9	68.6
E182A	69.6	70.1	69.2	70.6
S285D	66.8	67.2	67.0	67.3
S285Y	67.8	68.3	68.2	68.2

B ΔT_m of PqsE-HJ complexes

	DMSO	HJ1	HJ2	HJ5
WT	0.00	3.56	0.74	1.82
E280A	0.00	2.28	0.61	1.21
E182W	0.00	0.98	0.48	2.05
E182W/E280A	0.00	2.58	0.20	2.12
S285W	0.00	0.07	0.07	0.74
S285A	0.00	0.81	0.61	1.35
E182A	0.00	0.50	-0.47	1.00
S285D	0.00	0.35	0.14	0.48
S285Y	0.00	0.54	0.41	0.40

Fig S2: T_m measurements for PqsE(WT) and PqsE variants in the presence of HJ derivatives. PqsE (2 μM) was incubated with each molecule (100 μM) and the resulting T_m was measured by DSF. A) All average T_m values determined from three independent experiments performed in triplicate. Color scale is set with the lowest value in the whole data set (66.5) as white and the highest value (75.5) as green. B) The ΔT_m was determined by subtracting the DMSO-treated protein T_m from each inhibitor-treated protein T_m. As in A, the color scale is set with the lowest value in the whole data set (-0.47) as white and the highest value (3.56) as green.

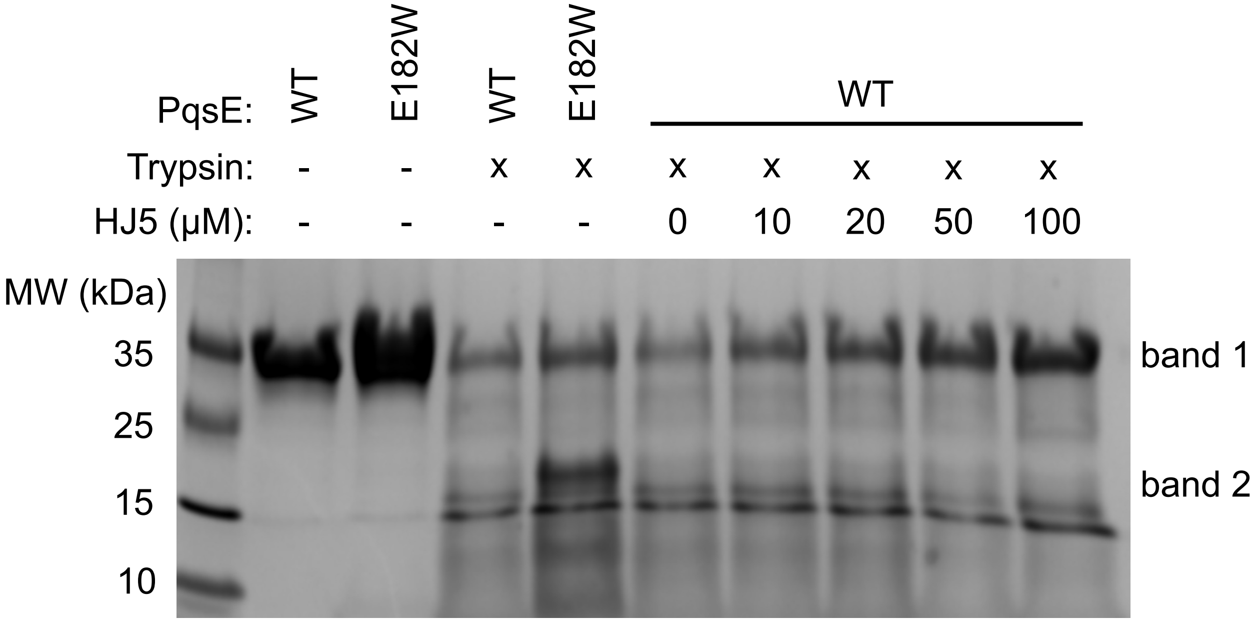

Fig S3: Additional partial proteolysis experiment. SDS-PAGE image of PqsE proteins partially digested with trypsin in the presence of varying concentrations of HJ5. In the first two lanes after the ladder, PqsE(WT) and PqsE(E182W) were not exposed to partial proteolysis by trypsin. The undigested protein runs as a single ~34 kDa band (band 1). The next two lanes show the banding patterns of PqsE(WT) and PqsE(E182W) produced from a 10-min incubation with trypsin. The presence of a distinct band at ~20 kDa (band 2) is diagnostic of the conformational shift induced by the E182W substitution. In the next 5 lanes, PqsE(WT) was incubated with the indicated concentrations of HJ5 prior to digestion by trypsin for 10 min. Band weight calculations were performed on this gel image and used along with the gel image in Fig 6a to generate the data shown in Fig 6b.

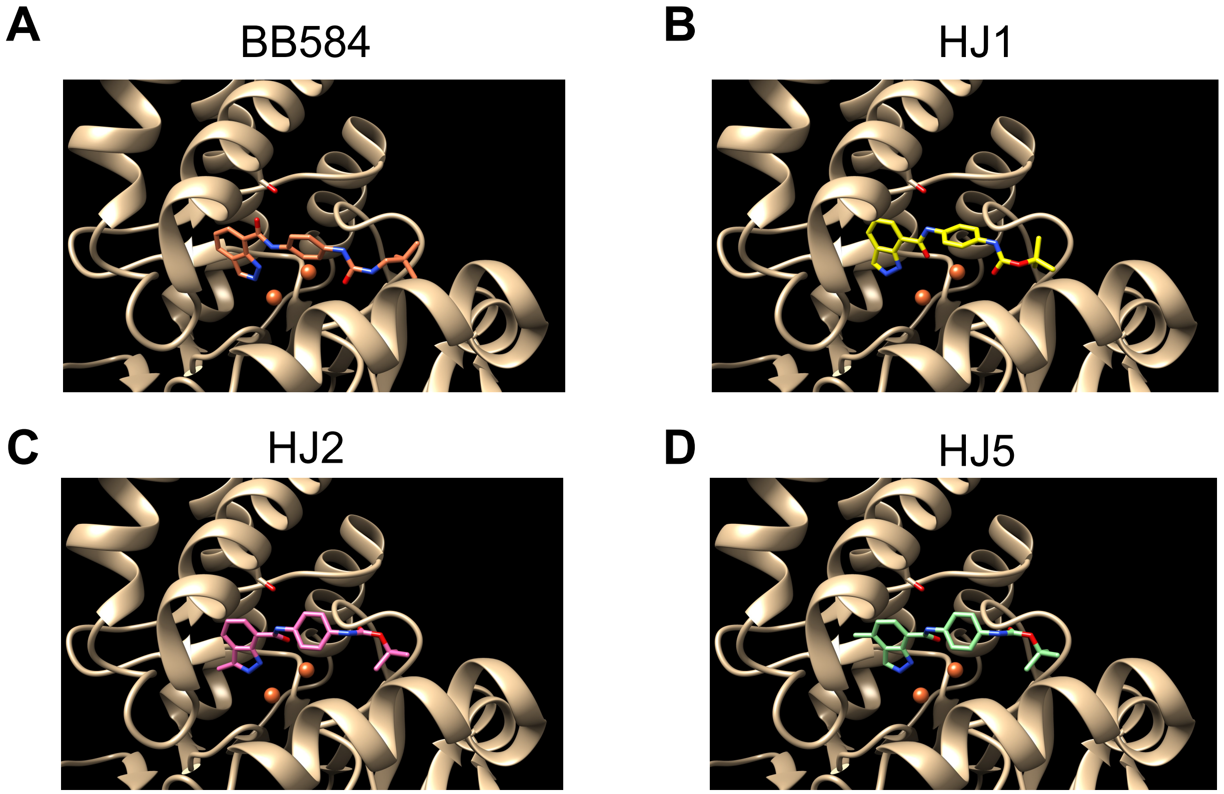

Fig S4: Docking of new small molecule BB584 derivatives in active site of PqsE. A) Structure of BB584 bound to PqsE as comparison to docking analysis (PDB: 7TZA). The protein backbone is depicted as a tan cartoon with the S285 sidechain shown as sticks. BB584 is shown with the stick representation with carbons in orange, nitrogens in blue and oxygens in red. The two iron atoms in the active site are shown as orange spheres. New HJ series molecules were docked to the PqsE structure in panel A with the ligand, BB584, removed. All docking simulations were performed with AutoDock Vina and visualized with Chimera. The docked ligands are shown with the carbons of HJ1 in yellow (B), carbons of HJ2 in pink (C), and carbons of HJ5 in green (D). HJ2 was experimentally determined to lose binding capability while HJ1 and HJ5 do retain binding. Also noted, the docking analysis predicted inversion of the amide groups upon binding of the HJ molecules compared to the BB584-bound structure. This could affect the actual orientation of the methyl substitutions on the indazole rings of HJ2 and HJ5, which will need to be investigated by structural methods.

Supplementary Tables

**Strain**	**Description**	**Reference**
UCBPP-PA14	PA14 *P. aeruginosa* Wildtype	Laboratory stock
SM776	*E. coli* BL21 (DE3) pET28b-6xHis-pqsE(WT)	22
IT39	*E. coli* BL21 (DE3) pET28b-6xHis-pqsE(S285A)	18
IT55	*E. coli* BL21 (DE3) pET28b-6xHis-pqsE(S285W)	18
IT105	PA14 *P. aeruginosa* Δ*pqsE*	21
IT169	PA14 *P. aeruginosa pqsE(S285W)*	This study

Table S1. Strains used in this study.
